# Clinical Characteristics and Outcomes of Hospitalized Malaria Patients in Rural Madagascar

**DOI:** 10.3390/jcm15062389

**Published:** 2026-03-20

**Authors:** Daniel Kasprowicz, Krzysztof Korzeniewski, Wanesa Wilczyńska

**Affiliations:** 1 Clinique Médicale Beyzym, Manerinerina RN6, Ambatoboeny District, Manerinerina 403, Madagascar; daniel.kasprowicz@icloud.com; 2 Department of Epidemiology and Tropical Medicine, Military Institute of Medicine–National Research Institute, 04-141 Warsaw, Poland; kkorzeniewski@wim.mil.pl

**Keywords:** severe malaria, hospital admission, *Plasmodium falciparum*, length of stay, rural health systems

## Abstract

**Background/Objectives:** Malaria remains a major cause of hospitalization in rural Madagascar, yet data on in-hospital clinical presentation, management, and patient outcomes remain limited. **Methods:** We conducted a three-year retrospective study (2023–2025) at a rural district hospital in Ambatoboeny, Madagascar, including patients of all ages hospitalized with malaria confirmed by rapid diagnostic testing and microscopy. Sociodemographic, clinical, laboratory, and treatment data were extracted from routine records. Length of hospital stay (LOS) was analyzed continuously and categorized as ≤2, 3–4, or ≥5 days. Seasonal admission patterns and factors associated with LOS were assessed using chi-square or Fisher’s exact tests, and associations with rainfall seasonality were explored using Spearman’s correlation. **Results:** Among 134 hospitalized patients, median age was 15 years (interquartile range (IQR) 7–25) and 52.2% were female. *Plasmodium falciparum* predominated (94.0%), while mixed-species infections were identified in 6.0% of cases; 20.1% of cases were classified as severe malaria, including 10.4% with cerebral malaria. Co-infections were frequent (52.2%), most commonly *Schistosoma haematobium* infection (14.2%) and typhoid fever (12.7%). Intravenous artesunate was initiated in 97.8% of patients; all received paracetamol and 94.8% received intravenous fluids. Median LOS was 2 days (IQR 2–3); 12.7% had prolonged hospitalization (≥5 days). Prolonged LOS was significantly associated with cerebral malaria, high parasitemia (≥5%), blood transfusion, and age < 15 years (all *p* ≤ 0.034), while co-infection and nutritional status were not. **Conclusions:** Hospitalized malaria in rural Madagascar presents with heterogeneous clinical phenotypes and a high burden of co-infections. Prolonged LOS is primarily driven by markers of severe disease and supportive care requirements, underscoring the need for early severity recognition and resource planning in low-resource hospitals.

## 1. Introduction

Malaria remains a major cause of hospital admission in sub-Saharan Africa, which continues to bear the overwhelming burden of global malaria morbidity and mortality [1]. Despite this substantial disease burden, standardized malaria treatment guidelines issued by the World Health Organization (WHO) are widely accessible and provide clear recommendations for the management of both uncomplicated and severe malaria, including in peripheral and resource-limited healthcare settings [2]. The introduction of parenteral artesunate as first-line therapy for severe malaria has markedly improved survival and overall clinical outcomes; however, recent studies suggest that recovery trajectories among hospitalized patients remain heterogeneous, with considerable variability in length of hospital stay (LOS) despite guideline-concordant treatment [3]. Emerging evidence indicates that factors such as disease severity at presentation, neurological and gastrointestinal manifestations, anemia, high parasite density, and the presence of co-infections may substantially influence in-hospital clinical course and prolong LOS. In malaria-endemic settings, co-infections are common due to overlapping ecological exposure to parasitic, bacterial, and protozoal pathogens, as well as shared environmental and socioeconomic determinants of infectious diseases [4,5]. These observations underscore that, beyond antimalarial efficacy alone, patient-level clinical characteristics and early manifestations play a critical role in determining hospitalization dynamics and outcomes in rural, resource-limited settings.

The aim of this study was to describe the clinical characteristics, management, and hospital course of patients hospitalized with malaria in a rural district hospital in Madagascar over a three-year period (2023–2025). This study was designed to describe in-hospital clinical care and outcomes, rather than to assess malaria epidemiology at the population level. By providing detailed clinical data from a rural hospital setting, the study may also contribute to improved understanding of hospitalization patterns and support health system planning in malaria-endemic regions of Madagascar.

## 2. Materials and Methods

### 2.1. Study Design and Setting

This retrospective observational study included all patients presenting with malaria to the Clinique Médicale BEYZYM, a secondary-level referral hospital, between January 2023 and December 2025. The facility is located at the border of the Ambatoboeny and Mampikony districts, serving populations from both districts within the Mahajanga Province, Madagascar.

During the study period, malaria diagnostics and clinical management were conducted in accordance with national malaria control guidelines. The hospital provides inpatient and outpatient care and functions as a referral center for surrounding rural, nurse-led health posts.

All patients presenting with malaria-compatible symptoms (e.g., fever, headache, chills, or influenza-like illness) were routinely screened using rapid diagnostic tests (RDTs). Over the three-year study period, 2456 malaria RDTs were performed, of which 282 were positive.

Patients with uncomplicated malaria and mild clinical presentation (*n* = 139) were managed on an outpatient basis and excluded from further analysis.

### 2.2. Study Population

**Figure 1:**
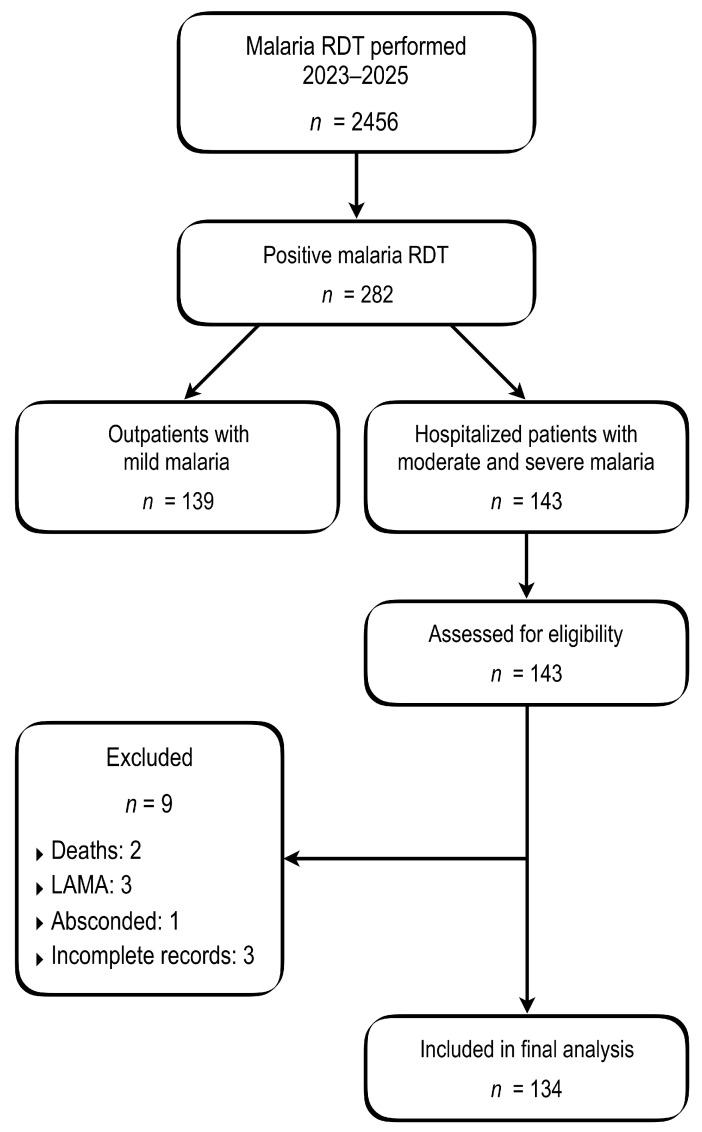
Flow diagram of patient selection and inclusion in the study.

Of the 143 patients hospitalized with moderate or severe malaria, nine patients were excluded because complete clinical and hospitalization data required for analysis were unavailable. These exclusions comprised early deaths occurring within six hours of admission, which precluded completion of diagnostic and laboratory evaluation (*n* = 2), leave against medical advice (LAMA) (*n* = 3), unauthorized departure (*n* = 1), and incomplete hospitalization records (*n* = 3). The final analytical cohort therefore comprised 134 hospitalized patients (Figure 1).

Inclusion criteria were a positive malaria RDTs followed by microscopic confirmation using both thick blood film and thin blood smear examination. Patients of all age groups were eligible for inclusion. For analytical purposes, patients were categorized into four age groups in accordance with WHO classifications: 0–4 years, 5–14 years, 15–49 years, and ≥50 years.

### 2.3. Case Definition and Classification

Case definitions and severity classification were based on WHO malaria guidelines in effect during the study period (2022–2024), with clinical assessment adapted to local diagnostic availability [2].

Mixed-species malaria infections were classified as such when more than one *Plasmodium* species was identified by microscopy and/or RDT. All cases were retained in the analysis according to their final classification.

LOS was defined as the number of days from the date of hospital admission to the date of discharge. For analytical purposes, LOS was additionally categorized as short (≤2 days), intermediate (3–4 days), or prolonged (≥5 days).

### 2.4. Data Sources and Data Collection

Data were collected retrospectively from multiple hospital-based sources. These included individual hospitalization records, nursing registers documenting vital signs, laboratory archives containing diagnostic test results, and hospital monthly reports summarizing the number of malaria tests performed as well as inpatient and outpatient malaria cases.

Clinical, laboratory, and treatment-related data were extracted manually. Data extraction was performed by the study authors in collaboration with key hospital personnel, including nursing, laboratory, and medical leadership staff.

All data were anonymized prior to analysis. Patient identifiers were removed, and the dataset used for statistical analyses contained no information enabling individual identification.

In the absence of locally measured monthly precipitation data for the Boeny region, long-term observed climatological averages (1991–2020) obtained from the World Bank Group Climate Change Knowledge Portal were used to characterize typical seasonal rainfall patterns and aggregated at the monthly level for exploratory analyses [6].

### 2.5. Variables

#### 2.5.1. Sociodemographic Variables

Sociodemographic data were extracted from standardized hospitalization records, which routinely include age, sex, place of residence, and occupation. Occupational status was analyzed only among patients aged ≥ 15 years.

#### 2.5.2. Clinical Variables

Clinical data were obtained from admission records. The primary reason for hospital presentation (chief complaint) was recorded in a dedicated field on the hospitalization form, while additional symptoms were documented by the admitting physician after clinical assessment.

Vital signs and anthropometric measurements at admission were collected by trained nursing staff using certified equipment. These included body temperature, oxygen saturation, weight, and height. Blood pressure was routinely recorded at admission in patients aged ≥ 15 years. Only baseline (admission) measurements were used for analyses.

Nutritional status was assessed at admission. In children, nutritional status was determined using weight-for-age indices (WFA) [7,8,9], while in older patients it was assessed using body mass index (BMI).

#### 2.5.3. Disease Classification and Coding

All diagnoses recorded at admission, including malaria co-infections and non-malaria co-morbidities, were retrospectively categorized using the International Classification of Diseases, 11th Revision (ICD-11) [10]. Each condition was assigned an ICD-11 code based on the final clinical diagnosis documented in the medical records. Co-infections were defined as the presence of one or more infectious diseases diagnosed concurrently with malaria during hospitalization. Multiple co-occurring infections were identified and grouped accordingly for descriptive analyses.

#### 2.5.4. Laboratory Variables

Laboratory investigations were performed by qualified laboratory personnel using equipment routinely employed in hospital clinical practice. Venous blood samples were collected using vacuum systems into EDTA tubes and plain tubes (without anticoagulant). EDTA blood was used for complete blood count (CBC), which was analyzed using an automated hematology analyzer (Mindray M-30, Mindray Bio-Medical Electronics Co., Shenzhen, China). Plain tubes were used for biochemical and serological analyses, which were performed using an automated chemistry analyzer (Sinnowa BS-3000, Sinnowa Medical Science & Technology Co., Nanjing, China). Reagents and diagnostic kits for biochemical and serological testing were supplied by Cypress Diagnostics (Langdorp, Belgium) and used according to the manufacturer’s instructions.

Microscopic malaria diagnostics were performed using thick and thin blood smears using May–Grünwald stain. Thin smears were used for *Plasmodium* species identification and estimation of parasite density (parasitemia, %), while thick films were used to confirm parasitemia.

#### 2.5.5. Treatment and Supportive Care Variables

Treatment variables were extracted from medication charts and hospitalization notes. Antimalarial therapy was categorized by route of administration (intravenous—IV, intramuscular—IM, or per os—PO) and by regimen, including parenteral artesunate or artemether and oral artemisinin-based combination therapy (ACT) used as step-down treatment.

Supportive care variables included administration of antipyretics (paracetamol), IV fluids, blood transfusion, antiemetics, iron supplementation, anthelmintics, and anticonvulsants. Antibiotic therapy was recorded by drug class (e.g., third-generation cephalosporins, penicillins, nitroimidazoles, aminoglycosides), rather than by individual agents.

### 2.6. Statistical Analysis

Descriptive statistics were used to summarize sociodemographic, clinical, laboratory, and treatment-related variables. Categorical variables were reported as frequencies and percentages, while continuous variables were summarized using medians and interquartile ranges (IQRs), as appropriate.

Raw clinical and laboratory data were initially transcribed and organized using Apple Numbers (version 14.1) for data cleaning, verification, and preliminary calculations.

To assess seasonal variation in malaria-related hospital admissions, a chi-square goodness-of-fit test (χ^2^) was performed to evaluate deviation from an equal monthly distribution, with effect size estimated using Cramér’s V. Associations between monthly malaria-related hospitalizations and precipitation levels were evaluated using Spearman’s rank correlation coefficient (ρ), given the non-normal distribution of variables. Correlation analyses were conducted for concurrent monthly precipitation as well as for precipitation lagged by one and two months.

Associations between LOS categories and clinical or demographic factors were assessed using χ^2^. When appropriate, Fisher’s exact test was considered for comparisons involving small cell counts.

Statistical significance was defined as a two-sided *p*-value < 0.05. All statistical analyses were performed using Python (version 3.11) with the SciPy statistical library (scipy.stats).

### 2.7. Ethical Considerations

This study was conducted in accordance with the principles of the Declaration of Helsinki. Authorization to conduct the study was obtained from the District Public Health Service (SSPD/27/07AB/2024), which provides administrative oversight for the study area, as well as ethical approval from the Hospital Supervisory Committee of the clinic. Given the retrospective design of the study and the use of routinely collected, fully anonymized clinical data, the requirement for individual informed consent was waived.

All data were fully de-identified prior to analysis, and no information enabling individual patient identification was included in the study dataset.

## 3. Results

### 3.1. Sociodemographic Characteristics of the Study Population

A total of 134 patients hospitalized with confirmed malaria were included in the analysis. The median age of the study population was 15 years (IQR: 7–25; range: 1–75 years), with nearly half of the patients belonging to the 15–49-year age group. Slightly more than half of the cohort were female, and the vast majority of patients originated from the Ambatoboeny District.

Normal nutritional status predominated at admission, although a substantial proportion of patients were underweight or malnourished. Occupational status was analyzed only among individuals aged ≥ 15 years, with subsistence farming and manual labour being the most frequently reported occupations. Detailed sociodemographic characteristics of the study population are presented in (Table 1).

### 3.2. Clinical Profile of Hospitalized Malaria Patients

#### 3.2.1. Clinical Presentation at Admission

Fever was the most common primary reason for hospital admission, reported in 62.7% of patients. Other frequent reasons for seeking hospital care included vomiting (12.7%), impaired consciousness or unconsciousness (6.0%), headache (5.2%), and abdominal pain (3.7%). Less common reasons for admission comprised generalized weakness and other non-specific complaints.

#### 3.2.2. Clinical Symptoms at Presentation

Fever was the most prevalent symptom, followed by vomiting, headache, abdominal pain, myalgia, chills, and diarrhea. Neurological manifestations, including confusion, seizures, and coma, were observed in a subset of patients. Several individuals presented with more than one symptom concurrently.

**Figure 2:**
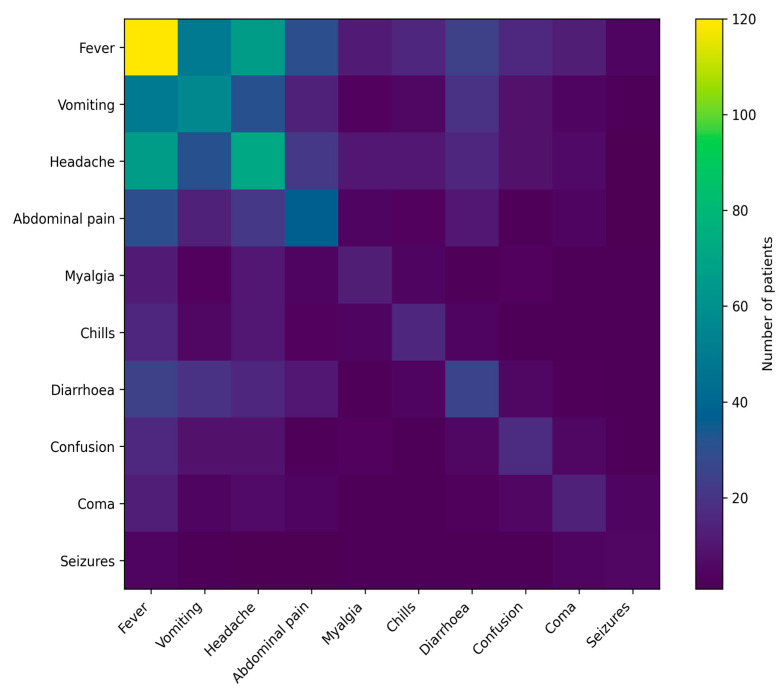
Heatmap showing co-occurrence of clinical symptoms at presentation among hospitalized malaria patients. Color intensity represents the number of patients in whom symptoms occurred concurrently.

A heatmap analysis highlighted patterns of symptom co-occurrence, demonstrating that fever frequently accompanied both gastrointestinal and neurological manifestations (Figure 2).

#### 3.2.3. Laboratory Findings at Admission

Baseline laboratory and vital parameters at admission, restricted to variables available for all patients (*n* = 134), are summarized in (Table 2).

#### 3.2.4. Co-Morbidities and Co-Infections

Co-infections were frequently observed among hospitalized malaria patients. Overall, 52.2% of individuals presented with at least one concurrent infectious disease. The most commonly identified co-infections were *Schistosoma haematobium* infection (14.2%), typhoid fever (12.7%), and *Salmonella enteritis* (9.7%), followed by other parasitic and bacterial infections (Table 3, Panel A). Human immunodeficiency virus (HIV) infection was documented in 3 patients (2.2%).

Non-malaria co-morbidities were relatively uncommon, affecting 5.2% of patients. These were predominantly chronic conditions, with primary hypertension being the most frequently reported (3.0%), while other co-morbidities occurred sporadically (Table 3, Panel B).

A notable proportion of patients exhibited a multi-infectious burden. The most frequent co-occurring infectious patterns involved *S. haematobium* infection combined with enteric pathogens, particularly *Salmonella enteritis*. Overall, 11.9% of patients presented with two or more concurrent co-infections, and 5.2% harbored multiple parasitic infections simultaneously (Table 3, Panel C).

### 3.3. Malaria-Specific Characteristics

Among the 134 hospitalized malaria cases, *Plasmodium falciparum* was the predominant species, identified in 126 patients (94.0%). Mixed-species malaria was detected in 8 cases (6.0%). RDTs occasionally demonstrated pan-*Plasmodium* antigen reactivity in addition to *P. falciparum*-specific bands.

Parasitemia levels ranged from 1% to 9%, with a median value of 2% (IQR: 2–3). The majority of patients (75.2%) presented with parasitemia levels of ≤3%, reflecting low-to-moderate parasite density at admission. High parasitemia (≥5%) was observed in 19 patients (14.2%), representing a clinically relevant subgroup and contributing to the classification of severe malaria.

Overall, cerebral malaria was diagnosed in 14 patients (10.4%), and malaria in pregnancy was identified in 9 cases (6.7%). Based on clinical presentation and available laboratory findings, 27 patients (20.1%) were classified as having severe malaria, whereas 107 patients (79.9%) were categorized as having moderate malaria.

In addition, seven patients (5.2%) were referred to the hospital due to clinical deterioration following inadequate prior treatment, consisting of IM quinine administered in rural nurse-led health posts.

#### Seasonality of Hospital Admissions

**Figure 3:**
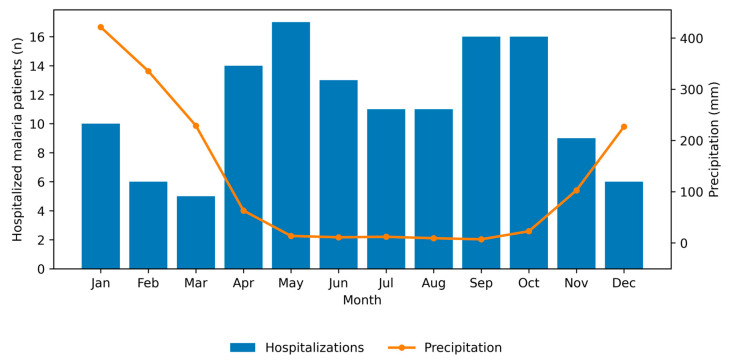
Monthly distribution of malaria-related hospitalizations and mean monthly precipitation. Hospitalization data were aggregated for the period 2023–2025. Precipitation data represent long-term average monthly rainfall (1991–2020) obtained from the World Bank Group Climate Change Knowledge Portal for the Boeny region, Madagascar.

The monthly distribution of malaria-related hospitalizations showed visible seasonal variation, with higher proportions observed during the post-rainy months, particularly in May (12.7%), September (11.9%), and October (11.9%), and lower admission counts during February–March and December. The monthly distribution of malaria-related hospitalizations and corresponding precipitation levels are shown in (Figure 3).

χ^2^ test did not demonstrate a statistically significant deviation from an equal monthly distribution (χ^2^ = 16.99, df = 11, *p* = 0.108; Cramér’s V = 0.11), indicating a small overall effect size for seasonality of hospital admissions.

Correlation analysis revealed a significant negative association between monthly hospitalizations and concurrent precipitation levels (Spearman’s ρ = −0.67, *p* = 0.017). A similar trend was observed for precipitation lagged by one month (ρ = −0.58, *p* = 0.061), although this association did not reach statistical significance. No association was observed for precipitation lagged by two months.

### 3.4. Treatment and Supportive Care

Antimalarial therapy was initiated predominantly via IV route (97.8%), with IM administration used rarely (1.5%) and oral-only initiation documented in a single case (0.7%). IV treatment consisted exclusively of artesunate, while IM therapy was administered as artemether. PO antimalarial therapy was provided as artemether + lumefantrine. Notably, quinine was not used in any patient.

PO ACT was used during hospitalization in 61.9% of patients, most commonly as step-down therapy following parenteral treatment (IV artesunate plus oral ACT: 60.4%). The median duration of antimalarial treatment was 4 days (IQR 4–5; range 2–10).

Supportive care was frequently required. All patients received paracetamol (100.0%). IV fluids were administered in 94.8% of patients for a median of 1 day (IQR 1–2; range 1–10). Antibiotics were prescribed in 43.3% of cases. The most commonly used antibiotic classes were third-generation cephalosporins (27.6%), penicillins (14.9%), nitroimidazoles (14.2%), and aminoglycosides (9.7%). Antiemetics were used in 44.0% of patients, iron supplementation in 50.7%, anthelmintics in 20.9% and anticonvulsants in 8.2%.

Blood transfusion was indicated in 15.7% of patients and performed in 12.7%, with a median of 1 unit transfused (IQR 1–2; range 1–4). Nasogastric feeding was required in 5.2% of cases and urinary catheterization in 6.0%.

Overall, antimalarial treatment was standardized and guideline-concordant, while supportive care requirements reflected the clinical severity of hospitalized cases.

### 3.5. Factors Associated with LOS

The median LOS was 2 days (IQR: 2–3; range: 1–22 days). Most patients experienced short hospitalizations, with 54.5% discharged within 2 days. An intermediate LOS (3–4 days) was observed in 32.8% of cases, while prolonged hospitalization (≥5 days) occurred in 12.7% of patients. These prolonged stays represented a small but clinically distinct subgroup requiring extended inpatient care.

Several clinical factors were significantly associated with LOS (Table 4). Prolonged hospitalization was more frequent among patients with severe malaria, particularly those presenting with cerebral involvement, high parasite density, and requiring blood transfusion (*p* < 0.001). In contrast, patients with moderate malaria were predominantly discharged within a short period.

Younger age (<15 years) was also associated with longer hospital stay. No significant association was observed between length of hospitalization and malaria in pregnancy, presence of any co-infection, or nutritional status at admission.

## 4. Discussion

Our findings confirm that malaria among hospitalized patients in a rural endemic setting rarely conforms to the classical clinical triad of fever, chills, and sweating, but rather presents as a heterogeneous syndrome characterized by overlapping systemic, gastrointestinal, and neurological manifestations. Although fever remained the most common presenting symptom, a substantial proportion of patients exhibited symptom constellations dominated by vomiting, abdominal pain, diarrhea, or altered mental status, underscoring the limited diagnostic utility of isolated symptoms in routine clinical practice. Similar observations have been increasingly reported across endemic regions, where malaria frequently mimics other acute febrile or gastrointestinal illnesses and challenges symptom-based diagnostic heuristics [9,11,12,13].

The prominent gastrointestinal phenotype observed in our cohort, particularly the frequent co-occurrence of fever with vomiting and abdominal complaints, aligns with recent hospital-based and emergency medicine studies emphasizing the non-specific and systemic nature of malaria presentation [11,14,15,16]. In endemic settings characterized by a high background prevalence of enteric infections and parasitic diseases, gastrointestinal symptoms may dominate the clinical picture and obscure malaria as the primary diagnosis [13,15,17]. This overlap likely reflects both shared inflammatory pathways and the frequent coexistence of concurrent infections, reinforcing the need to interpret gastrointestinal manifestations as an integral component of malaria rather than as atypical or secondary features.

Neurological manifestations in our study, including confusion, seizures, and coma, tended to cluster rather than occur in isolation, suggesting a distinct clinical phenotype associated with severe disease. This pattern is consistent with studies of cerebral malaria demonstrating that neurological symptoms often emerge as a constellation reflecting widespread microvascular dysfunction, neuroinflammation, and endothelial activation rather than isolated central nervous system involvement [18,19,20,21]. Importantly, the frequency and configuration of neurological signs vary across age groups and epidemiological contexts, with pediatric cohorts often displaying higher rates of seizures and coma compared with mixed or adult populations [18,19]. Our findings extend this observation by demonstrating that even in a predominantly non-pediatric cohort, neurological symptom clustering remains a key marker of severe clinical presentation.

Beyond discrete symptom categories, the observed co-occurrence patterns highlight malaria as a multidimensional clinical syndrome rather than a single-trajectory disease. Several recent analyses have proposed syndromic or cluster-based approaches to malaria symptomatology, showing that combinations of fever with respiratory, gastrointestinal, or neurological symptoms may better reflect real-world clinical presentations and improve risk stratification [9,12,22,23,24]. In this context, our heatmap-based analysis provides complementary evidence that symptom clustering may offer clinically relevant insights, particularly in resource-limited hospital settings where access to advanced diagnostics is constrained.

Taken together, the growing body of literature describing heterogeneous symptom profiles suggests that deviations from the classical triad should not be interpreted as atypical, but rather as context-dependent expressions of disease shaped by host immunity, parasite characteristics, co-infections, and health system factors [16,21,25,26,27]. Our findings support this evolving conceptualization and emphasize that recognizing symptom patterns—rather than relying on canonical descriptions—may be critical for timely diagnosis and appropriate management of hospitalized malaria patients in endemic, resource-constrained environments.

Among baseline laboratory abnormalities, thrombocytopenia and anemia were the most prominent findings in our cohort, reflecting well-recognized hematological consequences of acute malaria. Thrombocytopenia, frequently observed at admission, has been consistently reported across diverse endemic settings and is thought to result from a combination of peripheral platelet destruction, splenic sequestration, immune-mediated mechanisms, and platelet consumption during systemic inflammation [28,29,30,31]. While often transient, severe thrombocytopenia represents a clinically relevant challenge in low-resource settings such as rural Madagascar, where platelet concentrates are not routinely available and management relies largely on supportive care and close clinical monitoring rather than targeted transfusion therapy [29,32]. Anemia was also common at presentation and likely reflects both acute hemolysis related to *Plasmodium* infection and pre-existing vulnerabilities in endemic populations, including nutritional deficiencies and chronic parasitic exposure [26,30]. Although neither thrombocytopenia nor anemia alone dictated clinical outcomes in our study, their high prevalence underscores the importance of early laboratory assessment in hospitalized malaria patients and highlights the intersection between biological disease severity and structural limitations of care in resource-constrained health systems.

Analysis of monthly malaria-related hospitalizations revealed a non-uniform temporal distribution, with higher admission frequencies observed predominantly during post-rainy months rather than during periods of peak precipitation. Although the chi-square test did not demonstrate a statistically significant deviation from an equal monthly distribution, correlation analyses identified a significant negative association between hospitalizations and concurrent rainfall, as well as a weaker, non-significant trend with rainfall that lagged by one month, while no association was observed with a two-month lag. This pattern suggests that malaria-related hospitalizations in our setting are temporally displaced from peak rainfall, likely reflecting delayed effects of vector proliferation, parasite transmission dynamics, and progression from infection to clinically severe disease requiring inpatient care. Similar post-rainy or lagged seasonal patterns have been reported across diverse endemic regions, where malaria incidence or hospitalization peaks often occur weeks to months after maximal rainfall rather than during the rainy season itself [33,34,35,36,37]. Studies from Madagascar and comparable ecological settings further highlight substantial regional heterogeneity in malaria seasonality, influenced by local climate, vector ecology, population immunity, and healthcare access, underscoring that hospital-based malaria data capture not only transmission intensity but also delayed and severity-dependent manifestations of disease [38,39,40,41]. Together, these findings emphasize that rainfall–malaria relationships are complex and context-dependent, and that hospitalization patterns should be interpreted as downstream indicators of transmission rather than direct proxies of seasonal malaria incidence.

Beyond ecological drivers of malaria transmission, temporal patterns of hospital admissions in rural agrarian settings may also reflect socioeconomic constraints affecting healthcare-seeking behavior. In subsistence-farming communities across sub-Saharan Africa and Madagascar, seasonal fluctuations in food security, household income, and agricultural workload have been shown to influence both nutritional status and access to healthcare services [42,43,44]. Studies from rural Madagascar demonstrate that periods preceding harvest are characterized by heightened food insecurity and limited financial resources, whereas post-harvest periods are associated with improved household food availability and liquidity, potentially facilitating access to medical care [43,44]. Similar seasonal dynamics have been described in West African settings, where malaria prevalence, antibiotic use, and nutritional indicators fluctuate across agricultural cycles rather than strictly mirroring rainfall patterns [42]. Consequently, hospital-based malaria admission data may capture not only delayed effects of transmission intensity but also seasonally mediated barriers to care, leading to clustering of severe cases during periods when healthcare becomes economically accessible. This context underscores that temporal variation in malaria-related hospitalizations reflects a complex interplay between transmission ecology, nutritional vulnerability, and structural access to care, rather than transmission intensity alone.

In our cohort, LOS was primarily driven by markers of disease severity rather than by the presence of co-infections or variability in antimalarial therapy. Prolonged hospitalization was significantly associated with cerebral malaria, high parasite density, and the need for blood transfusion, while younger age showed a modest but statistically significant association with longer LOS. These findings are consistent with multiple hospital-based studies showing that neurological involvement, severe anemia requiring transfusion, and high parasitemia are among the strongest predictors of extended LOS and increased resource utilization in malaria patients [13,45,46,47,48]. Notably, despite a high prevalence of co-infections in our study population, their presence was not independently associated with prolonged hospitalization, a finding that contrasts with some reports linking bacterial or parasitic co-infections to extended LOS but aligns with others suggesting that timely and effective antimalarial treatment may mitigate their impact on inpatient course [49,50,51,52]. Importantly, all patients in our cohort received guideline-concordant artemisinin-based therapy, resulting in a relatively short median LOS despite the inclusion of severe cases, underscoring the central role of effective antimalarial treatment in shortening hospitalization duration [53,54,55,56]. In this context, the requirement for blood transfusion emerged not only as a marker of biological disease severity but also as a proxy for health system burden, particularly in resource-limited settings where transfusion capacity is constrained and transfusion practices may directly influence hospitalization duration [56,57]. Collectively, these findings highlight that LOS in hospitalized malaria patients reflects an interplay between clinical severity, supportive care requirements, and system-level capacity, rather than the mere presence of concurrent infections.

Nutritional status was also evaluated in our cohort but was not significantly associated with hospitalization duration. Previous studies suggest that the relationship between malaria and nutritional status is complex and heterogeneous, with some investigations reporting increased susceptibility or disease severity among malnourished individuals, while others have not demonstrated a consistent association between baseline nutritional status and malaria-related clinical outcomes [58,59,60].

This study highlights the clinical and operational challenges of managing hospitalized malaria patients in rural endemic settings and underscores the importance of context-specific interpretation of symptom patterns, laboratory abnormalities, and hospitalization dynamics.

### 4.1. Implications for Public Health

From a public health perspective, these findings highlight the importance of strengthening early recognition and management of severe malaria in rural health systems. Although most hospitalized patients experienced short hospital stays, a small subset with severe disease—particularly those with cerebral malaria, high parasitemia, or requiring blood transfusion—accounted for a disproportionate share of inpatient resource use. Improving early diagnosis and timely referral from peripheral nurse-led posts may therefore reduce the burden of severe presentations at district hospitals. In rural endemic settings, this may include not only educational interventions but also logistical and organizational measures aimed at strengthening the decision-making capacity of frontline healthcare workers, particularly nurses who often provide primary care in remote communities. Targeted training programs focusing on early identification of severe malaria and prompt referral to higher-level facilities may therefore represent an important component of health system strengthening. In addition, improving access to basic rapid diagnostic tools at peripheral health posts—particularly those facilitating early detection of severe anemia—and training in standardized clinical assessment methods, such as consciousness evaluation scales, may further support early recognition of severe disease. The observed temporal patterns of hospitalization also suggest that hospital-based malaria services should anticipate seasonal fluctuations and adjust clinical resources accordingly. Finally, the high prevalence of concurrent infections underscores the need for integrated diagnostic and treatment approaches in endemic settings where malaria frequently coexists with other infectious diseases. Together, these observations support the value of strengthening clinical capacity and resource planning in rural hospitals managing malaria in endemic regions.

### 4.2. Study Limitations

This study has several limitations. The retrospective design precluded prospective standardization and the collection of additional laboratory tests beyond routine clinical practice. The absence of a standardized questionnaire limited the assessment of non-clinical determinants of LOS. As a single-center, hospital-based cohort conducted in a rural district hospital, the study is subject to referral and selection bias, and the findings do not represent population-level malaria epidemiology. In addition, some variables were assessed only when clinically indicated, introducing potential information bias and under-ascertainment of co-infections and co-morbidities. Finally, LOS may have been influenced by health system and patient-level factors, and outcomes were limited to the in-hospital course without post-discharge follow-up.

## 5. Conclusions

Malaria in hospitalized patients from rural endemic settings presents with heterogeneous clinical manifestations that frequently extend beyond the classical triad, highlighting the diagnostic relevance of gastrointestinal and neurological symptoms. Our findings suggest that hospitalization dynamics in such settings are shaped not only by transmission ecology but also by delayed seasonal effects and healthcare access patterns. Prolonged hospitalization appears to be primarily driven by markers of disease severity and the need for supportive care rather than by the presence of concurrent infections. These observations underline the importance of early recognition of severe malaria and adequate resource planning in rural hospitals managing malaria in endemic regions.

## Figures and Tables

**Table 1:** Sociodemographic characteristics of hospitalized malaria patients (*n* = 134).

Characteristic	*n*	%
**Age (years)**		
Median (IQR)	15 (7–25)	—
Range (min–max)	1–75	—
**Age groups**		
0–4 years	20	14.9
5–14 years	44	32.8
15–49 years	63	47.0
≥50 years	7	5.2
**Sex**		
Female	70	52.2
Male	64	47.8
**Place of residence**		
Ambatoboeny District	113	84.3
Mampikony District	17	12.7
Maevatanana District	2	1.5
Other regions	2	1.5
**Nutritional status**		
Normal	88	65.7
Underweight	31	23.1
Malnutrition	11	8.2
Overweight	4	3.0
**Occupation (age ≥ 15 years; *n* = 68)**		
Rice cultivator	37	54.4
Student	17	25.0
Seller	7	10.3
Miner	3	4.4
Teacher	2	2.9
Housekeeper	2	2.9

**Table 2:** Baseline laboratory and vital parameters at hospital admission (*n* = 134).

Parameter	Median (IQR)	Min–Max
Hemoglobin (g/dL)	10.2 (8.8–11.8)	4.6–17.3
Platelets (×10^9^/L)	142.5 (97.2–159.0)	15.0–535.0
White blood cells (×10^9^/L)	6.9 (5.3–9.5)	2.6–23.7
Eosinophils (%)	4.0 (1.7–8.8)	0.9–35.0
C-reactive protein (mg/L)	12.0 (6.0–24.0)	3.0–98.0
Sodium (mmol/L)	138.0 (134.0–138.0)	123.0–156.0
Potassium (mmol/L)	3.6 (3.5–4.2)	2.4–5.8
Body temperature (°C)	36.7 (36.5–37.5)	35.3–40.1
Heart rate (beats/min)	103 (85–120)	60–187
Oxygen saturation SpO_2_ (%)	99 (98–99)	85–99

**Table 3:** Multi-burdens of infectious diseases among hospitalized malaria patients (*n* = 134).

Panel A. Co-Infections			
Co-Infection	ICD-11 Code	*n*	%
Any co-infection (≥1)	—	70	52.2
Schistosomiasis due to *S. haematobium*	1F86.0	19	14.2
Typhoid fever	1A07	17	12.7
*Salmonella enteritis*	1A09.0	13	9.7
Unspecified protozoal disease	1F5Z	11	8.2
Unspecified parasitic diseases	1G2Z	8	6.0
Bacterial pneumonia, unspecified	CA40.0Z	5	3.7
Bacterial vaginosis	MF3A	3	2.2
HIV disease associated with malaria	1C61	3	2.2
Urinary tract infection	GC08	2	1.5
Sepsis without septic shock	1G40	2	1.5
Other (each *n* = 1) *	—	6	4.5
**Panel B. Non-malaria co-morbidities**			
Non-malaria co-morbidity	ICD-11	*n*	%
Any non-malaria co-morbidity	—	7	5.2
Essential hypertension	BA00	4	3.0
Sickle cell disease (without crisis)	3A51.1	1	0.7
Kwashiorkor	5B52	1	0.7
Gastric ulcer	DA60	1	0.7
**Panel C. Most frequent co-occurring infectious conditions**			
Co-occurring conditions	ICD-11	*n*	%
Schistosomiasis due to *S. haematobium* + *Salmonella* enteritis	1F86.0 + 1A09.0	4	3.0
Typhoid fever + intestinal parasitic disease	1A07 + 1G2Z	2	1.5
Intestinal parasitic disease + protozoal disease	1G2Z + 1F5Z	2	1.5
≥2 parasitic co-infections **	multiple	7	5.2
≥2 co-infections (any type)	multiple	16	11.9

* Includes gonorrhoeae, trichomoniasis, meningitis (unspecified), acute nasopharyngitis, deep bacterial folliculitis/skin abscess, and toxoplasmosis. ** Parasitic co-infections include schistosomiasis, intestinal parasitic disease, protozoal disease, and toxoplasmosis.

**Table 4:** Factors associated with length of hospital stay among hospitalized malaria patients.

Variable	LOS ≤ 2 Days	LOS 3–4 Days	LOS ≥ 5 Days	*p*-Value
Cerebral malaria	2	7	9	<0.001
Malaria in pregnancy	4	4	1	0.74
High parasitemia (≥5%)	6	5	8	<0.001
Blood transfusion	0	9	8	<0.001
Any co-infection	34	25	11	0.31
Non-normal nutritional status	21	17	8	0.27
Age < 15 years	29	25	12	0.034

## Data Availability

The data presented in this study are available upon request from the corresponding author.

## References

[1] World Health Organization World Malaria Report 2025. https://www.who.int/teams/global-malaria-programme/reports/world-malaria-report-2025.

[2] World Health Organization WHO Guidelines for Malaria. https://www.who.int/publications/i/item/guidelines-for-malaria.

[3] Gesase S., Onyamboko M., Fanello C., Abdul O., Kayembe D.K., Bakomba S.B., Minja D.T.R., Nzambiwishe B.K., Ekombolo P.E., Malabeja A. (2026). Single-step versus conventional injectable artesunate for severe malaria in children: An open label, non-inferiority randomized clinical trial, Democratic Republic of the Congo and United Republic of Tanzania. Bull. World Health Organ..

[4] Menezes R.A.O., Gomes M.D.S.M., Mendes A.M., Couto Á.A.R.A., Nacher M., Pimenta T.S., Sousa A.C.P., Baptista A.R.S., Jesus M.I., Enk M.J. (2018). Enteroparasite and vivax malaria co-infection on the Brazil-French Guiana border: Epidemiological, haematological and immunological aspects. PLoS ONE.

[5] Nacher M. (2011). Interactions between worms and malaria: Good worms or bad worms?. Malar. J..

[6] World Bank Group Madagascar: Climate Data—Historical. https://climateknowledgeportal.worldbank.org/country/madagascar/climate-data-historical.

[7] World Health Organization Growth Reference Data for 5–19 Years: Weight-for-Age. https://www.who.int/tools/growth-reference-data-for-5to19-years/indicators/weight-for-age-5to10-years.

[8] World Health Organization Child Growth Standards: Weight-for-Age. https://www.who.int/tools/child-growth-standards/standards/weight-for-age.

[9] Muwonge H., Akugizibwe R., Bbosa G., Damani A.M., Nakidde B., Mugahi R., Byakika-Kibwika P. (2025). Syndromic presentation by patients with malaria and respiratory symptoms in a malaria-endemic urban-and-peri-urban setting. BMC Res. Notes.

[10] World Health Organization International Classification of Diseases, 11th Revision (ICD-11). https://icd.who.int/.

[11] Long B., MacDonald A., Liang S.Y., Brady W.J., Koyfman A., Gottlieb M., Chavez S. (2024). Malaria: A focused review for the emergency medicine clinician. Am. J. Emerg. Med..

[12] Kwinda W., Mudzweda A.D., Luhalima T. (2026). Experiences of people diagnosed with malaria regarding signs and symptoms at a selected village in the Vhembe District, Limpopo province, South Africa. Curationis.

[13] Lompo P., Tahita M.C., Sorgho H., Kaboré W., Kazieng A., Nana A.C.B., Natama H.M., Bonkoungou I.J.O., Barro N., Tinto H. (2021). Pathogens associated with acute diarrhea and comorbidity with malaria among children under five years old in rural Burkina Faso. Pan Afr. Med. J..

[14] Gehlawat V.K., Arya V., Kaushik J.S., Gathwala G. (2013). Clinical spectrum and treatment outcome of severe malaria caused by *Plasmodium vivax* in 18 children from northern India. Pathog. Glob. Health.

[15] Sugg K.N., Mpata F.W., Humes M.D., Marachto D.A., Rajan R., Winch P.J. (2026). Positive tests are all alike, every negative test is negative in its own way: Lack of confidence in negative malaria rapid diagnostic tests in the Democratic Republic of the Congo. Malar. J..

[16] Acharya J., Harwani D. (2022). Changing pattern of severe manifestations of *Plasmodium falciparum* and *Plasmodium vivax* malaria: A retrospective study from Bikaner, India. J. Vector Borne Dis..

[17] McQueen A., Mannheim J. (2025). Diagnosis and management of imported malaria in the emergency department. Pediatr. Emerg. Care.

[18] Jegede T.O., Oseni S.B., Okeniyi J.A.O., Kuti B.P., Adegoke S.A., Salau Q.A., Bello E.O., Jegede T.P., Kareem A.J., Oyelami O. (2024). Pattern of clinical and laboratory presentation of cerebral malaria among children in Nigeria. J. Glob. Infect. Dis..

[19] Sharma I., Kataria P., Das J. (2024). Cerebral malaria pathogenesis: Dissecting the role of CD4+ and CD8+ T-cells as major effectors in disease pathology. Int. Rev. Immunol..

[20] Wu X., Qin N., Yi F., Wang J., Yan X., Wang L. (2024). Cerebral malaria presenting as nonconvulsive status epilepticus: A case report. Malar. J..

[21] Edassery A., Meher A.K., Gupta V., Rodriguez R. (2024). Clinico-epidemiological profiles and outcome of severe malaria in children under-five in the tribal area of Kalahandi, Odisha. Indian J. Med. Res..

[22] Beniwal P., Joshi J., Kaur S. (2025). A comprehensive review of cerebral malaria. J. Parasit. Dis..

[23] Akech S., Chepkirui M., Ogero M., Agweyu A., Irimu G., English M., Snow R.W. (2020). The Clinical Profile of Severe Pediatric Malaria in an Area Targeted for Routine RTS,S/AS01 Malaria Vaccination in Western Kenya. Clin. Infect. Dis..

[24] Mawili-Mboumba D.P., Batchy Ognagosso F.B., M’Bondoukwé N.P., Ngomo J.M.N., Ditombi B.C.M., Agbanrin A.A., Nymane T., Ngondza B.P., Mouandza R.M., Mihindou C.J. (2025). Hospital attendance, malaria prevalence and self-medication with an antimalarial drug before and after the start of COVID-19 pandemic in a sentinel site for malaria surveillance in Gabon. Malar. J..

[25] Martins Y.C., Murin P.J., Siqueira-E-Silva B.N., Daniel-Ribeiro C.T. (2025). The relationship between malaria and pain: A mini-review. Am. J. Trop. Med. Hyg..

[26] Song T., Chen J., Huang L., Gan W., Yin H., Jiang J., He T., Huang H., Hu X. (2016). Should we abandon quinine plus antibiotic for treating uncomplicated *falciparum* malaria? A systematic review and meta-analysis of randomized controlled trials. Parasitol. Res..

[27] Zayet S., Hagenkötter B., Quadrio I., Gendrin V., Klopfenstein T. (2024). Severe malaria with neurological manifestations: What contribution of neurofilament light chain?. J. Infect. Dis..

[28] Gurumurthy G., Gurumurthy J., Gurumurthy S., Reynolds L., Swan D., Thachil J. (2026). Clinical characteristics of thrombocytopenia in tropical diseases and management in resource-limited settings. Res. Pract. Thromb. Haemost..

[29] Balsa-Vázquez J., Rubio-Muñoz J.M., Duvignaud A., Alcedo S., Pérez de Ayala A., Salas-Coronas J., Elía-López M., Cattaneo P., Arévalo-Cañas C., García-Bujalance S. (2026). Comparison of routine clinical profiles of patients with imported *P. falciparum* malaria and co-infection with *Plasmodium ovale wallikeri* or *Plasmodium ovale curtisi*. Acta Trop..

[30] Nunthanasup N., Palasuwan A., Palasuwan D., Combes V. (2025). Interactions between *Plasmodium falciparum*-infected red blood cells and their extracellular vesicles with megakaryocytes: Implications for platelet-like particle formation. Malar. J..

[31] Ndatumuremyi J., Kagimbangabo J.M.V., Nshimiyimana I., Gaspard B., Sendegeya J.P., Nizeyimana V., Byukusenge C., Nsanzabera C. (2025). Prevalence and factors associated with severe thrombocytopenia among hospitalized patients with malaria attending Kigeme District Hospital, Southern Province, Rwanda: Cross-sectional study. Malar. J..

[32] Özer D., Çavuş İ., Özbilgin A. (2025). Artesunate and severe malaria: Importance of treatment and laboratory monitoring. Mikrobiyol. Bul..

[33] Rabia R., Ullah W. (2026). Spatial Distribution, seasonal dynamics and molecular confirmation of malaria in District Malakand, Khyber Pakhtunkhwa, Pakistan. Trans. R. Soc. Trop. Med. Hyg..

[34] Djedanem M., Yacoubou M.A.Y., Zakari A., Issa I., Yahaya M., Zaneidou M., Mody I., Onana D.B., Testa J., Jambou R. (2026). Burden of malaria among children aged 5-10 years in the Sahelian area: Do we need to adapt seasonal malaria chemoprophylaxis. BMC Infect. Dis..

[35] Thomson M.C., Mason S.J., Phindela T., Connor S.J. (2005). Rainfall and sea surface temperature monitoring for malaria early warning in Botswana. Am. J. Trop. Med. Hyg..

[36] Kapolo P., Chiepa B., Mbewe R.B., Kapumba B., Kambewa E., Kaunga L., Coleman S., Chirombo J., Mzilahowa T., Jones C.M. (2026). Repeated biannual cross-sectional surveys in primary schools set baseline seasonal and spatial surveillance for malaria and schistosomiasis in the Shire Valley Transformation Programme (SVTP), Malawi. Curr. Res. Parasitol. Vector Borne Dis..

[37] Mussa A., Rodrigues M., Enosse S.M., Cook L., Bunce L. (2026). Lessons from using SALAMA (DIGIT HCM-health campaign management platform) to implement and optimize seasonal malaria chemoprevention in Nampula, Mozambique. Oxf. Open Digit. Health.

[38] Nguyen M., Howes R.E., Lucas T.C.D., Battle K.E., Cameron E., Gibson H.S., Rozier J., Keddie S., Collins E., Arambepola R. (2020). Mapping malaria seasonality in Madagascar using health facility data. BMC Med..

[39] Kang S.Y., Battle K.E., Gibson H.S., Ratsimbasoa A., Randrianarivelojosia M., Ramboarina S., Zimmerman P.A., Weiss D.J., Cameron E., Gething P.W. (2018). Spatio-temporal mapping of Madagascar’s Malaria Indicator Survey results to assess *Plasmodium falciparum* endemicity trends between 2011 and 2016. BMC Med..

[40] Arambepola R., Keddie S.H., Collins E.L., Twohig K.A., Amratia P., Bertozzi-Villa A., Chestnutt E.G., Harris J., Millar J., Rozier J. (2020). Spatiotemporal mapping of malaria prevalence in Madagascar using routine surveillance and health survey data. Sci. Rep..

[41] Randriamihaja M., Randrianjatovo T.M., Evans M.V., Ihantamalala F.A., Herbreteau V., Révillion C., Delaitre E., Catry T., Garchitorena A. (2025). Monitoring individual rice field flooding dynamics over a large scale to improve mosquito surveillance and control. Malar. J..

[42] Roberts S.A., Brabin L., Tinto H., Gies S., Diallo S., Brabin B. (2021). Seasonal patterns of malaria and nutrition in adolescents in Burkina Faso. BMC Public Health.

[43] Raholiarimanana F., Rakotomanana H., Ishida A. (2023). Livestock and child dietary diversity in Madagascar. Children.

[44] Herrera J.P., Rabezara J.Y., Ravelomanantsoa N.A.F., Metz M., France C., Owens A., Oender M., Nunn C.L., Kramer R.A. (2021). Food insecurity related to agricultural practices and household characteristics in rural communities of northeast Madagascar. Food Secur..

[45] Gallet S., Dard C., Bailly S., Thellier M., Houze S., Pelloux H., Epaulard O. (2024). Length of stay in at-risk areas and time to malaria attack on return. Infect. Dis. Now.

[46] Ikhurionan P.E., Bell N.V.T., Ofovwe G.E. (2024). Emergency blood transfusion outcomes in children in Sierra Leone. Pediatr. Emerg. Care.

[47] Moffitt C.A., Olupot-Olupot P., Onen J.W., O’Brien N. (2023). Adherence to severe malaria treatment guidelines in Uganda. Malar. J..

[48] Machini B., Achia T.N.O., Kipruto H., Amboko B., Chesang J. (2022). Factors associated with hospital stay in suspected malaria patients in Kenya. BMJ Open.

[49] Huang L., Jin H., Zhang H., Liu J., Shi X., Kang X., Zeng Y., Wang L. (2022). Factors associated with prolonged hospital stay of imported malaria cases in China. BMC Infect. Dis..

[50] Ahmed M.A.A., Musa I.R., Mahgoub H.M., Al-Nafeesah A., Al-Wutayd O., Adam I. (2022). Outcomes and Predictors of Pediatric Medical Admissions at Gadarif Hospital in Eastern Sudan. Front. Pediatr..

[51] Watts C., Atieli H., Alacapa J., Lee M.-C., Zhou G., Githeko A., Yan G., Wiseman V. (2021). Rethinking the economic costs of hospitalization for malaria: Accounting for the comorbidities of malaria patients in western Kenya. Malar. J..

[52] Hoffmeister B. (2021). Factors associated with prolonged hospital stay in imported falciparum malaria. Microorganisms.

[53] Chentsov V.B., Tokmalaev A.K., Kozhevnikova G.M., Baranova A.M., Vdovina E.T., Emerole K.C. (2020). Intensive care treatment of severe *Plasmodium falciparum* malaria. J. Trop. Med..

[54] Sikora S.A., Poespoprodjo J.R., Kenangalem E., Lampah D.A., Sugiarto P., Laksono I.S., Ahmad R.A., Murhandarwati E.E.H. (2019). Intravenous artesunate plus oral dihydroartemisinin-piperaquine or intravenous quinine plus oral quinine for optimum treatment of severe malaria: Lesson learnt from a field hospital in Timika, Papua, Indonesia. Malar. J..

[55] Mathiba R.M., Mathivha L.R., Nethathe G.D. (2019). Artesunate versus quinine for severe malaria in ICU patients. S. Afr. J. Crit. Care.

[56] Maitland K., Olupot-Olupot P., Kiguli S., Chagaluka G., Alaroker F., Opoka R.O., Mpoya A., Engoru C., Nteziyaremye J., Mallewa M. (2019). Transfusion volume for children with severe anemia in Africa. N. Engl. J. Med..

[57] Maitland K., Kiguli S., Olupot-Olupot P., Engoru C., Mallewa M., Saramago Gonclaves P., Opoka R.O., Mpoya A., Alaroker F., Nteziyaremye J. (2019). Immediate Transfusion in African Children with Uncomplicated Severe Anemia. N. Engl. J. Med..

[58] Das D., Grais R.F., Okiro E.A., Stepniewska K., Mansoor R., van der Kam S., Terlouw D.J. (2018). Complex interactions between malaria and malnutrition: A systematic literature review. BMC Med..

[59] Oldenburg C.E., Guerin P.J., Berthé F., Grais R.F., Isanaka S. (2018). Malaria and nutritional status among children with severe acute malnutrition in Niger: A prospective cohort study. Clin. Infect. Dis..

[60] Caulfield L.E., Richard S.A., Black R.E. (2004). Undernutrition as an underlying cause of malaria morbidity and mortality in children less than five years old. Am. J. Trop. Med. Hyg..

